# Letter to editor: ‘respiratory – swallow coordination training using bimodal signal biofeedback for patients with post – stroke dysphagia: a randomized controlled trial’

**DOI:** 10.1080/07853890.2026.2626237

**Published:** 2026-02-05

**Authors:** Isha Goel, Subhasish Chatterjee, Mousumi Saha

**Affiliations:** Maharishi Markandeshwar Institute of Physiotherapy and Rehabilitation, MMDU, Ambala, India

Dear Editor

We read with considerable interest the randomized controlled trial by Wang et al. investigating biomodal signal biofeedback – based respiratory – swallow coordination training for post – stroke dysphagia, published in Annals of Medicine [1]. The trial addresses respiratory – swallow coordination, a significant and clinically understudied mechanism, and suggests a novel dual – signal biofeedback method. Although the findings are encouraging, there are several methodological and interpretive concerns that should be further explored to improve clinical translation.

First, the central novelty of the intervention lies in the integration of respiratory airflow and accelerometry – derived swallow signals. However, treatment fidelity and patient learning consistency were not sufficiently quantified. Although progression criteria were described across recognition, learning and mastery modules, objective reporting of inter – session variability, adherence rates or failed progression attempts are lacking [2]. Clearer reporting of training fidelity would improve repeatability and interpretability because biofeedback – based motor learning heavily relies on patient participation and feedback accuracy.

Second, only individuals within six months of their stroke – a time frame marked by significant spontaneous neuroplastic recovery – were included in the study population [3]. Although this is acknowledged by the authors, no analytical technique was used to differentiate between spontaneous recovery trajectories and intervention – specific effects. The inclusion of time – since – stroke as a covariate or stratification by stroke chronicity could have improved causal inference and made it clearer which patient subgroups are most likely to benefit from this intervention.

Third, results from the Penetration – Aspiration Scale and the Functional Oral Intake Scale showed statistically significant improvements, but effect sizes were not disclosed. In rehabilitation research, effect magnitude is crucial for judging clinical relevance beyond statistical significance [4]. Reporting effect sizes or responder analyses, particularly for patients with severe baseline aspiration risk – would better inform clinicians regarding the real – world impact of the intervention.

Finally, video fluoroscopic temporal and kinematic parameters showed limited between-group differences despite functional improvement. Important mechanistic questions are raised by this decoupling, such as whether the intervention only improves compensatory techniques rather than changing swallowing biomechanics. Theoretical clarity will benefit from explicitly discussing this distinction, as it will inform future refinements.

In summary, Wang et al. offer a novel and clinically applicable rehabilitation approach with promising initial data. Future work regarding both defining and interpreting the concepts of training fidelity, spontaneous recovery effects, magnitude of treatment effects and mechanistic interpretation, will continue to solidify the evidence base, allowing for broader confident implementation, into clinical practice with bimodal biofeedback assisted respiratory – swallowing coordination training.

## Data Availability

No dataset was created or examined for this study, so data sharing is not relevant.
